# Supplementary material for: Association of physical activity and sleep habits during pregnancy with autistic spectrum disorder in 3-year-old infants
Source: Commun Med (Lond). 2022 Apr 5;2:35. doi: 10.1038/s43856-022-00101-y (PMC9053216; doi:10.1038/s43856-022-00101-y)
Supplement: Supplementary file 1 — Reporting Summary [file 43856_2022_101_MOESM1_ESM.pdf]

nature research

Corresponding author(s): Seiichi Morokuma

Last updated by author(s): Feb 21, 2022

Reporting Summary

Nature Research wishes to improve the reproducibility of the work that we publish. This form provides structure for consistency and transparency in reporting. For further information on Nature Research policies, see our Editorial Policies and the Editorial Policy Checklist.

Statistics

For all statistical analyses, confirm that the following items are present in the figure legend, table legend, main text, or Methods section.

n/a	Confirmed	
☐	☒	The exact sample size (n) for each experimental group/condition, given as a discrete number and unit of measurement
☐	☒	A statement on whether measurements were taken from distinct samples or whether the same sample was measured repeatedly
☒	☐	The statistical test(s) used AND whether they are one- or two-sided<br>*Only common tests should be described solely by name; describe more complex techniques in the Methods section.*
☐	☒	A description of all covariates tested
☒	☐	A description of any assumptions or corrections, such as tests of normality and adjustment for multiple comparisons
☐	☒	A full description of the statistical parameters including central tendency (e.g. means) or other basic estimates (e.g. regression coefficient) AND variation (e.g. standard deviation) or associated estimates of uncertainty (e.g. confidence intervals)
☒	☐	For null hypothesis testing, the test statistic (e.g. F, t, r) with confidence intervals, effect sizes, degrees of freedom and P value noted<br>*Give P values as exact values whenever suitable.*
☒	☐	For Bayesian analysis, information on the choice of priors and Markov chain Monte Carlo settings
☒	☐	For hierarchical and complex designs, identification of the appropriate level for tests and full reporting of outcomes
☒	☐	Estimates of effect sizes (e.g. Cohen's d, Pearson's r), indicating how they were calculated

Our web collection on statistics for biologists contains articles on many of the points above.

Software and code

Policy information about availability of computer code

Data collection	We used a fixed dataset “jecs-ta-20190930,” which was made by the core center of the JECS study. This dataset is not open to the public.
Data analysis	Stata version 16

For manuscripts utilizing custom algorithms or software that are central to the research but not yet described in published literature, software must be made available to editors and reviewers. We strongly encourage code deposition in a community repository (e.g. GitHub). See the Nature Research guidelines for submitting code & software for further information.

Data

Policy information about availability of data

All manuscripts must include a data availability statement. This statement should provide the following information, where applicable:

- Accession codes, unique identifiers, or web links for publicly available datasets
- A list of figures that have associated raw data
- A description of any restrictions on data availability

Data are unsuitable for public deposition due to ethical restrictions and legal framework of Japan. It is prohibited by the Act on the Protection of Personal Information (Act No. 57 of 30 May 2003, amendment on 9 September 2015) to publicly deposit the data containing personal information. Ethical Guidelines for Medical and Health Research Involving Human Subjects enforced by the Japan Ministry of Education, Culture, Sports, Science and Technology and the Ministry of Health, Labour and Welfare also restricts the open sharing of the epidemiologic data. All inquiries about access to data should be sent to: jecs-en@nies.go.jp. The person responsible for handling enquiries sent to this e-mail address is Dr Shoji F. Nakayama, JECS Programme Office, National Institute for Environmental Studies.

Field-specific reporting

Please select the one below that is the best fit for your research. If you are not sure, read the appropriate sections before making your selection.

☒ Life sciences ☐ Behavioural & social sciences ☐ Ecological, evolutionary & environmental sciences

For a reference copy of the document with all sections, see nature.com/documents/nr-reporting-summary-flat.pdf

Life sciences study design

All studies must disclose on these points even when the disclosure is negative.

Sample size	We analyzed all JECS study participants who met the conditions of this study.<br>The subjects of analysis was 69,969 participants among all 103,060 participants in JECS study.
Data exclusions	We excluded 33,091 cases for the following reasons: previous participation in the study (n = 5,647), multiple fetuses (n = 948), miscarriage or stillbirth (n = 3,520), congenital anomaly or disease at 1 month of age (n = 3,550), missing information on maternal age at delivery (n = 11), delivery before 37 weeks or after 42 weeks of gestation (n = 4,454), and no response to all questions about maternal PA and sleep habits in the M-T1 and M-T2 (n = 751). Of the remaining 84,719 participants, 14,210 participants (17%) did not reply to the 3-year-old questionnaire.
Replication	When significant associations were found, we also adjusted paternal factors as sensitivity analysis.<br>The significant associations did not change from the result of the main analysis.
Randomization	We adjusted covariates in the multivariate analysis.
Blinding	Binding was not possible because of the design of this study.

Reporting for specific materials, systems and methods

We require information from authors about some types of materials, experimental systems and methods used in many studies. Here, indicate whether each material, system or method listed is relevant to your study. If you are not sure if a list item applies to your research, read the appropriate section before selecting a response.

Materials & experimental systems

n/a	Involved in the study	
☒	☐	Antibodies
☒	☐	Eukaryotic cell lines
☒	☐	Palaeontology and archaeology
☒	☐	Animals and other organisms
☐	☒	Human research participants
☒	☐	Clinical data
☒	☐	Dual use research of concern

Methods

n/a	Involved in the study	
☒	☐	ChIP-seq
☒	☐	Flow cytometry
☒	☐	MRI-based neuroimaging

Human research participants

Policy information about studies involving human research participants

Population characteristics	Japanese pregnant women who recruited to the JECS study between January 2011 and March 2014.
Recruitment	The face-to-face recruitment was conducted by the JECS research centers in each region throughout Japan.
Ethics oversight	The Japan Environment and Children's Study (JECS) protocol was reviewed and approved by the Ministry of the Environment's Institutional Review Board on Epidemiological Studies (no. 100910001) and by the Ethics Committees of all participating institutions.

Note that full information on the approval of the study protocol must also be provided in the manuscript.
