# Supplementary material for: Association of physical activity and sleep habits during pregnancy with autistic spectrum disorder in 3-year-old infants
Source: Commun Med (Lond). 2022 Apr 5;2:35. doi: 10.1038/s43856-022-00101-y (PMC9053216; doi:10.1038/s43856-022-00101-y)
Supplement: Supplementary file 2 — Supplementary Information [file 43856_2022_101_MOESM2_ESM.pdf]

Supplementary Table1. Association between physical activity and sleep during pregnancy and autism spectrum disorder in infants aged 3 years (parents-reported), adjusted with paternal factors, Japan Environment and Children's Study

During pregnancy	Analysis of association between maternal PA or sleep during pregnancy and infant ASD (Quotes from Table 1 and Table 2)						Analysis limited to cases with paternal data and adjusted for paternal factors					
	No. of participants	No. of outcomes		Multivariable model[a]			No. of participants	No. of outcomes		Multivariable model[b]		
			%	RR	95% CI				%	RR	95% CI	
**Physical activity**												
**0**	15,210	80	0.5	**0.91**	0.66	1.26	8,419	42	0.5	**0.85**	0.55	1.33
**Q1**	12,796	71	0.6	**Reference**			6,971	39	0.6	**Reference**		
**Q2**	12,281	48	0.4	**0.69**	0.48	0.99	6,436	24	0.4	**0.64**	0.38	1.06
**Q3**	13,382	67	0.5	**0.87**	0.62	1.21	6,954	32	0.5	**0.81**	0.50	1.29
**Q4**	12,839	43	0.3	**0.61**	0.42	0.90	6,585	19	0.3	**0.51**	0.29	0.91
**Sleep duration (hours)**												
**<6**	3,267	26	0.8	**1.87**	1.21	2.90	1,705	17	1.0	**2.39**	1.36	4.20
**6–7**	10,490	53	0.5	**1.20**	0.85	1.70	5,682	28	0.5	**1.21**	0.75	1.96
**7–8**	21,737	85	0.4	**Reference**			11,712	44	0.4	**Reference**		
**8–9**	19,739	83	0.4	**1.18**	0.87	1.60	10,505	37	0.4	**1.08**	0.69	1.69
**9–10**	9,801	47	0.5	**1.46**	1.03	2.11	4,985	21	0.4	**1.50**	0.88	2.55
**>10**	4,354	24	0.6	**1.56**	1.00	2.48	2,300	14	0.6	**1.91**	1.02	3.58

PA, physical activity; ASD, autistic spectrum disorder; CI, confidence interval; RR, risk ratio

[a]Adjusted for maternal age at delivery, smoking habits, alcohol consumption, pre-pregnancy body mass index, parity, infertility treatment, maternal educational background, history of depression, anxiety disorders, and schizophrenia, autistic traits, uterine infection, type of delivery, gestational age at birth, small for gestational age, infant sex, and feeding status.

[b]Adjusted for above factors and paternal factors (age, smoking, educational background, history of depression, anxiety disorders, schizophrenia, and autistic traits)
