# Supplementary material for: Association of physical activity and sleep habits during pregnancy with autistic spectrum disorder in 3-year-old infants
Source: Commun Med (Lond). 2022 Apr 5;2:35. doi: 10.1038/s43856-022-00101-y (PMC9053216; doi:10.1038/s43856-022-00101-y)
Supplement: Supplementary file 3 — Supplementary Data 1 [file 43856_2022_101_MOESM3_ESM.pdf]

Supplementary Data 1. Baseline characteristics of the participants of the Japan Environment and Children's Study (2011-2014)

	Physical activity during pregnancy						Sleep duration (hours) during pregnancy						
	No. of women*	0	Q1	Q2	Q3	Q4	No. of women*	<6	6–7	7–8	8–9	9–10	>10
		(%)	(%)	(%)	(%)	(%)		(%)	(%)	(%)	(%)	(%)	(%)
**Physical activity, median (IQR), METs-min/week**		0	198 (120–264)	495 (396–594)	1188 (984–1,440)	4,752 (2,970–7,920)							
**No. of women**	66,508	15,210	12,796	12,281	13,382	12,839	69,388	3,267	10,490	21,737	19,739	9,801	4,354
**Age at delivery (years)**													
< 25	5,562	7.2	7.7	7.4	8.9	10.8	5,796	9.5	7.4	6.9	7.4	9.4	19.3
25–29	18,203	24.7	25.3	27.3	28.3	31.7	18,979	25.0	26.4	27.0	27.1	28.3	32.3
30–34	24,126	36.4	37.5	36.2	36.5	34.9	25,148	33.0	34.6	36.9	37.6	37.6	30.5
≥ 35	18,617	31.8	29.6	29.1	26.4	22.6	19,465	32.5	31.7	29.2	28.0	24.7	18.0
**Smoking habits**													
Never smoked	40,296	61.4	62.3	62.1	61.7	55.7	42,049	56.6	62.3	62.3	61.2	58.9	53.2
Ex-smokers who quit before pregnancy	15,612	22.4	23.5	23.6	23.4	24.8	16,270	22.3	21.5	22.7	24.2	25.7	24.9
Smokers during early pregnancy	10,531	16.2	14.2	14.3	14.9	19.6	10,990	21.1	16.3	15.0	14.6	15.4	21.9
**Alcohol consumption**													
Never drank	22,988	37.1	34.6	33.9	33.3	33.5	24,065	33.6	33.5	34.4	35.2	35.5	35.7
Ex-drinkers who quit before pregnancy	11,993	16.9	17.9	18.1	18.4	19.2	12,500	17.2	15.9	17.2	18.3	20.6	21.0
Drinkers during early pregnancy	31,515	46.1	47.5	48.0	48.3	47.3	32,810	49.3	50.7	48.5	46.5	44.0	43.3
**Pre-pregnancy body mass index**													
< 18.5 kg/m^2	10,711	16.3	16.4	16.1	16.2	15.5	11,161	16.6	15.9	15.9	15.7	16.6	17.5

18.5–24.9 kg/m^2	49,388	74.1	74.8	74.4	74.5	73.8	51,519	73.1	74.7	74.8	74.7	73.3	72.5
≥ 25.0 kg/m^2	6,376	9.6	8.9	9.5	9.3	10.7	6,675	10.3	9.4	9.3	9.6	10.1	10.1
**Parity**													
0	30,004	43.1	46.1	48.2	47.3	42.2	31,218	58.0	59.6	50.9	37.4	29.0	43.7
≥ 1	36,273	56.9	53.9	51.8	52.7	57.8	37,931	42.1	40.4	49.1	62.6	71.0	56.3
**History of psychiatric disorders**													
Depression	1,908	2.8	2.9	2.8	3.1	2.8	1,980	4.8	3.1	2.6	2.6	2.9	3.5
Anxiety disorder	1,732	2.5	2.4	2.5	3.0	2.6	1,799	3.9	2.7	2.4	2.3	2.7	3.5
Schizophrenia	98	0.2	0.2	0.1	0.2	0.2	101	0.3	0.2	0.1	0.1	0.1	0.2
**Current history**													
Hypertensive disorders in pregnancy	1,779	2.9	2.7	2.5	2.5	2.6	1,855	3.7	3.1	2.8	2.4	2.4	2.1
Diabetes or gestational diabetes	1,969	2.7	3.1	3.4	3.0	2.7	2,072	3.6	3.1	3.1	2.7	2.9	3.2
Intrauterine infection	409	0.6	0.5	0.7	0.6	0.6	428	0.6	0.8	0.7	0.6	0.5	0.6
**Infertility treatment**													
No	61,817	93.0	92.3	91.9	93.0	94.7	64,506	93.0	91.2	92.3	93.4	94.8	95.4
Ovulation stimulation /artificial insemination with husband’s semen	2,570	3.8	4.1	4.3	3.9	3.3	2,665	3.9	4.6	4.2	3.7	3.0	2.8
Assisted reproductive technology	2,086	3.2	3.6	3.9	3.1	1.9	2,180	3.1	4.2	3.5	2.9	2.2	1.8
**Autistic traits**													
Negative (AQ-J-10 < 7)	64,716	97.1	97.6	97.3	97.6	97.6	67,518	96.3	97.6	97.5	97.7	97.1	96.7
Positive (AQ-J-10 ≥ 7)	1,721	2.9	2.4	2.7	2.4	2.5	1,794	3.7	2.4	2.5	2.3	2.9	3.3
**Educational background (years)**													
< 10	2,463	3.1	3.3	3.5	4.1	4.6	2,562	6.0	3.4	2.9	3.2	4.3	7.6
10–12	19,860	32.4	29.0	29.0	27.9	31.1	20,722	33.6	29.4	28.0	29.4	31.3	37.8

13–16	42,904	63.3	65.9	65.5	66.0	63.2	44,772	59.0	65.7	67.2	65.8	63.0	53.6
≥ 17	1,056	1.2	1.8	2.0	2.0	1.1	1,101	1.3	1.6	1.8	1.6	1.4	1.0
**Type of delivery**													
Vaginal	54,986	81.8	82.5	82.8	83.9	83.4	57,357	82.4	82.2	82.6	82.8	83.9	83.7
Cesarean	11,386	18.2	17.5	17.3	16.1	16.6	11,894	17.6	17.8	17.4	17.2	16.1	16.3
**Small for gestational age**													
No	61,540	93.0	93.2	92.6	92.7	92.8	64,198	93.1	92.7	92.7	93.1	92.8	93.0
Yes	4,734	7.0	6.8	7.4	7.3	7.2	4,948	6.9	7.3	7.3	7.0	7.2	7.0
**Gestational age (weeks)**													
37	6,384	10.8	9.6	9.0	8.9	9.5	6,648	9.4	9.0	9.4	10.0	9.7	9.9
38	15,246	23.2	23.3	22.4	22.5	23.2	15,935	23.1	22.6	22.8	23.1	23.9	21.7
39	19,699	29.0	29.6	30.2	30.1	29.4	20,541	28.4	28.7	29.4	30.0	30.5	30.4
40	18,669	27.0	28.0	28.0	28.9	28.6	19,473	28.1	28.7	28.6	27.6	27.0	28.2
41	6,510	10.0	9.6	10.4	9.6	9.3	6,791	11.0	11.1	9.8	9.3	8.9	9.8
**Infant sex**													
Boy	33,803	50.5	50.7	51.0	51.0	51.0	35,300	50.5	50.7	50.9	50.5	51.8	51.0
Girl	32,702	49.5	49.3	49.0	49.0	49.0	34,085	49.5	49.3	49.1	49.5	48.2	49.0
**Apgar score at 5 minutes**													
7–10	63,000	99.7	99.7	99.6	99.6	99.7	65,744	99.8	99.6	99.7	99.7	99.7	99.6
< 7	212	0.3	0.3	0.4	0.4	0.3	219	0.2	0.4	0.3	0.3	0.3	0.4
**Feeding status until 1 year old**													
Formula feeding	1,313	2.5	1.7	1.7	1.6	2.2	1,390	2.8	1.8	2.0	1.9	1.9	3.0
Partial breastfeeding	43,070	66.0	64.4	64.3	63.2	65.7	44,871	67.8	67.4	66.2	63.5	60.9	62.0
Exclusive breastfeeding	22,125	31.6	33.9	34.0	35.1	32.0	23,127	29.4	30.8	31.8	34.7	37.3	35.0

AQ-J-10, short form of the Autism Spectrum Quotient, Japanese version; MET, metabolic equivalent of a task; IQR, interquartile range

*Subgroup totals do not equal the overall number because of missing data.
