# Supplementary material for: Association of physical activity and sleep habits during pregnancy with autistic spectrum disorder in 3-year-old infants
Source: Commun Med (Lond). 2022 Apr 5;2:35. doi: 10.1038/s43856-022-00101-y (PMC9053216; doi:10.1038/s43856-022-00101-y)
Supplement: Supplementary file 4 — Supplementary Data 2 [file 43856_2022_101_MOESM4_ESM.pdf]

Supplementary Data 2. Comparison of backgrounds between the population analyzed and the population excluded from the analysis due to non-response to the C-3y questionnaire

		Population analyzed (n = 69,969)			Non-responders to the C-3y questionnaire (n = 14,210)	
		No. of women	(%)		No. of women	(%)
**Sleep duration during pregnancy (hours)**						
<6		3,267	4.7		859	6.3
6–7		10,490	15.1		1,955	14.3
7–8		21,737	31.3		4,022	29.4
8–9		19,739	28.5		3,806	27.8
9–10		9,801	14.1		1,915	14.0
>10		4,354	6.3		1,115	8.2
**Physical activity during pregnancy, median (IQR), METs-min/week**		495 (66-1,386)			495 (50-1,638)	
**Age at delivery (years)**						
< 25		5,884	8.4		2,500	17.6
25–29		19,166	27.4		4,213	29.7
30–34		25,325	36.2		4,475	31.5
≥ 35		19,594	28.0		3,022	21.3
**Smoking habits**						
Never smoked		42,356	60.6		6,589	46.5
Ex-smokers who quit before pregnancy		16,400	23.5		3,265	23.0
Smokers during early pregnancy		11,131	15.9		4,319	30.5

		Population analyzed (n = 69,969)			Non-responders to the C-3y questionnaire (n = 14,210)	
		No. of women	(%)		No. of women	(%)
**Alcohol consumption**						
Never drank		24,254	34.7		4,624	32.6
Ex-drinkers who quit before pregnancy		12,719	18.2		2,908	20.5
Drinkers during early pregnancy		32,981	47.2		6,655	46.9
**Pre-pregnancy body mass index**						
< 18.5 kg/m^2		11,261	16.1		2,289	16.1
18.5–24.9 kg/m^2		51,930	74.3		9,947	70.1
≥ 25.0 kg/m^2		6,743	9.6		1,955	13.8
**Parity**						
0		31,505	45.2		5,207	36.8
≥ 1		38,223	54.8		8,929	63.2
**History of psychiatric disorders**						
Depression		2,004	2.9		524	3.7
Anxiety disorder		1,819	2.6		530	3.8
Schizophrenia		102	0.2		47	0.3
**Current history**						
Hypertensive disorders in pregnancy		1,868	2.7		416	2.9
Diabetes or gestational diabetes		2,089	3.0		436	3.1
Intrauterine infection		429	0.6		94	0.7

		Population analyzed (n = 69,969)			Non-responders to the C-3y questionnaire (n = 14,210)	
		No. of women	(%)		No. of women	(%)
**Infertility treatment**						
No		65,051	93.0		13,644	96.2
Ovulation stimulation / artificial insemination with husband's semen		2,688	3.8		321	2.3
Assisted reproductive technology		2,193	3.1		225	1.6
**Autistic traits**						
Negative (AQ-J-10 ≤ 6)		67,784	97.4		13,356	97.1
Positive (AQ-J-10 > 6)		1,805	2.6		399	2.9
**Educational background (years)**						
< 10		2,591	3.7		1,360	9.9
10–12		20,827	30.0		5,252	38.3
13–16		44,899	64.7		6,963	50.8
≥ 17		1,103	1.6		132	1.0
**Type of delivery**						
Vaginal		57,828	82.8		11,593	81.8
Caesarean		11,994	17.2		2,585	18.2
**Small for gestational age**						
No		64,719	92.8		13,018	92.1
Yes		5,006	7.2		1,114	7.9

		Population analyzed (n = 69,969)			Non-responders to the C-3y questionnaire (n = 14,210)	
		No. of women	(%)		No. of women	(%)
**Gestational age (week)**						
37		6,712	9.6		1,445	10.2
38		16,069	23.0		3,386	23.8
39		20,723	29.6		4,146	29.2
40		19,625	28.1		3,922	27.6
41		6,840	9.8		1,311	9.2
**Infant sex**						
Boys		35,613	50.9		7,230	50.9
Girls		34,353	49.1		6,976	49.1
**Apgar score at 5 minutes**						
7–10		66,239	99.7		13,462	99.6
< 7		221	0.3		52	0.4
**Feeding status until 1-year-old**						
Formula feeding		1,413	2.0		286	2.0
Partial breastfeeding		45,266	64.7		11,404	80.3
Exclusive breastfeeding		23,290	33.3		2,520	17.7

C-3y, questionnaire administered at three years after delivery; IQR, interquartile range;

AQ-J-10, the short form of the Autism-Spectrum Quotient Japanese version

*Subgroup totals do not equal the overall number because of missing data.
