# Supplementary material for: Association of physical activity and sleep habits during pregnancy with autistic spectrum disorder in 3-year-old infants
Source: Commun Med (Lond). 2022 Apr 5;2:35. doi: 10.1038/s43856-022-00101-y (PMC9053216; doi:10.1038/s43856-022-00101-y)
Supplement: Supplementary file 5 — Description of Additional Supplementary Files [file 43856_2022_101_MOESM5_ESM.pdf]

Description of Additional Supplementary Files

File Name: Supplementary Data 1

Description: Baseline characteristics of the participants of the Japan Environment and Children's Study (2011-2014)

File Name: Supplementary Data 2

Description: Comparison of backgrounds between the population analyzed and the population excluded from the analysis due to non-response to the C-3y questionnaire
